# The effect of the cytoplasmic tail of influenza C virus CM2 protein on its biochemical properties and intracellular processing

**DOI:** 10.1016/j.bbrep.2015.07.001

**Published:** 2015-07-08

**Authors:** Yoshitaka Shimotai, Takanari Goto, Yoko Matsuzaki, Yasushi Muraki, Kanetsu Sugawara, Seiji Hongo

**Affiliations:** a Department of Infectious Diseases, Yamagata University Faculty of Medicine, 2-2-2 Iida-Nishi, Yamagata 990-9585, Japan; b Department of Microbiology, Iwate Medical University, 2-1-1 Nishitokuta, Yahaba, Iwate 028-3694, Japan

**Keywords:** Influenza C virus, CM2, Ion channel, Cytoplasmic tail, Biochemical property

## Abstract

CM2 is an integral membrane protein encoded by the influenza C virus M gene. To examine the effects of the cytoplasmic tail of CM2 on its biochemical properties, deletion and substitution mutations were introduced into CM2 cytoplasmic tail at residues 47–115, and the expressed CM2 mutants were investigated. Although the cytoplasmic tail is not essential for the oligomerization of CM2, it may affect the degree of oligomerization. The residues 47–48, 67–69, 73–90 and 113–115 were all required for the proper expression of CM2. Pulse-chase experiments suggest that residues 47–48, 67–69, 73–75 and 79–87 stabilize CM2, thereby affecting CM2 expression. The C-terminal region at residues 61–115 is not essential for CM2 transport to the cell surface, and a 14-amino-acid, but not an 11-amino-acid, cytoplasmic tail is sufficient for the cell surface expression of CM2. These results suggest that either certain amino acid sequences or the length of the CM2 cytoplasmic tail are necessary for the proper conformational maturation, stability, expression level and intracellular transport of CM2.

## Highlights

• The cytoplasmic tail modulates the efficiency of CM2 oligomerization.
• The residues 47–48, 67–69, 73–90 and 113–115 are required for the proper expression of CM2.
• The residues 47–48, 67–69, 73–75 and 79–87 stabilize CM2.
• Cytoplasmic 14 amino acids are sufficient for the cell surface expression of CM2.

## 1. Introduction

The influenza C virus genome consists of seven single-stranded RNA segments of negative polarity [1]. The spliced and unspliced mRNAs from RNA segment 6 (M gene) of influenza C virus encode the 242-amino-acid (aa) matrix (M1) protein, and a 374-aa protein, designated P42, which contains an additional 132 amino acids from the C-terminus of M1 [2], [3], [4]. P42 is cleaved by signal peptidase at the C-terminal side of Ala259 to generate CM2, composed of the C-terminal 115 amino acids, and M1′, composed of the N-terminal 259 amino acids [5], [6].

CM2 is an integral membrane protein that is oriented in membranes with 23-aa N-terminal extracellular, 23-aa transmembrane, and 69-aa C-terminal cytoplasmic domains [7], [8]. CM2 is modified post-translationally by N-glycosylation at Asn11, which converts non-glycosylated CM2_0_ (Mr~16 kDa) into CM2a (Mr~18 kDa) [7], [8]. The processing of the carbohydrate chain from a high-mannose type to a complex type converts CM2a into CM2b with heterogeneous electrophoretic mobility (Mr~22–30 kDa) [7]. CM2 is also modified by palmitoylation at Cys65 and by phosphorylation at Ser78 and Ser103 [7], [9], [10]. Furthermore, CM2 forms dimers and tetramers by intermolecular disulfide bonds at Cys1, Cys6 and Cys20 [7], [8], [10]. CM2 is transported to the cell surface [7], [8], [10] and is also incorporated into virions [7].

The whole-cell currents of *Xenopus laevis* oocytes expressing CM2 demonstrated that CM2 forms a voltage-activated ion channel permeable to chloride ions [11]. When CM2 was co-expressed with a pH-sensitive influenza A virus hemagglutinin (HA), CM2 protected the HA against low pH-induced conformational change in the trans-Golgi network, indicating that CM2 can elevate trans-Golgi pH via proton permeability [12].

The M2 protein of influenza A virus possesses proton ion channel activity, and is thought to conduct protons from the endosomes into the interior of the virus to disrupt interactions between the vRNPs and M1, facilitating uncoating [1]. In addition, M2 plays a role in infectious virion production, through facilitation of the efficient packaging of genome segments into virions, as well as in virion morphology [1]. Influenza B virus BM2 protein also possesses proton channel activity [13] and is involved in genome packaging [14]. As the biochemical properties of CM2 are very similar to those of M2 [7], [8] and, like M2 and BM2, CM2 appears to possess proton permeability [12], it is reasonable to assume that CM2 may play a role in influenza C virus replication. Therefore, we examined the effect of CM2 on influenza C virus replication and demonstrated that CM2 plays a role in the packaging of the reporter gene into influenza C virus-like particles (VLPs) as well as in the uncoating of the VLPs, suggesting that CM2 is essential for virus replication [15]. The loss of sites for N-glycosylation and disulfide bond formation reduced virus growth, suggesting that these post-translational modifications in the extracellular domain of CM2 are required for efficient virus replication [16], [17]. In contrast, no inhibition in growth was observed in viruses lacking a palmitoylation site in the cytoplasmic region of CM2, suggesting that the palmitoylation of CM2 does not affect virus replication [18]. The cytoplasmic domains of influenza A virus M2 and influenza B virus BM2 play a role in virion morphology and genome packaging [19], [20], [21], [22]. However, except for palmitoylation, the effect of the cytoplasmic tail of CM2 on influenza C virus replication has not yet been investigated. In this study, we examined whether the cytoplasmic tail of CM2 affects its biochemical properties and intracellular functions prior to an analysis of the effect of the CM2 cytoplasmic tail on influenza C virus replication. To this end, deletion and substitution mutations were introduced into the cytoplasmic tail of CM2, and analyses of the expressed CM2 mutants indicated that the cytoplasmic residues are involved in the expression level, oligomerization, stability and intracellular transport of CM2.

## 2. Materials and methods

### 2.1. Ethics statement

The present study was approved by the Safety Committee for Gene Recombination Experiments of Yamagata University.

### 2.2. Cells and antibodies

COS-1 cells were maintained in Dulbecco's modified Eagle's medium with 10% fetal bovine serum [4], [5], [10]. Monoclonal antibody against FLAG epitope (monoclonal anti-FLAG M2) was purchased from SIGMA. Rabbit antiserum against the GST fusion protein containing the CM2 protein (anti-GST/CM2 serum) was prepared as described previously [3].

### 2.3. Construction of CM2 cytoplasmic tail mutants

A plasmid expressing FLAG-tagged wild-type CM2 (FLAG-CM2 1–115) was constructed as follows: cDNA from the M gene of influenza C/Ann Arbor/1/50 covering positions 803–1150, which encode residues 1–115 of CM2, was first amplified using a sense primer, +EcoRI-FLAG-M803 (5′-GACGAATTCACC**ATGGACTACAAGGACGACGATGACAAG**TGCAATCTAAAGACC-3′ [EcoRI site, underlined; the nucleotides encoding the FLAG tag, bold, were followed by the sequence corresponding to positions 803–817]), an antisense primer, −NotI-M1150 (5′-TATGCGGCCGC**TTA**AATTTCAAAAATACC-3′ [NotI site, underlined; the stop codon, bold, was followed by the sequence corresponding to positions 1150–1133]), and a PolI plasmid expressing segment 6 vRNA of influenza C/Ann Arbor/1/50, pPolI/M, as a template [17], [18]. The PCR products were digested with EcoRI and NotI, and then cloned into the EcoRI and NotI sites of the transient expression vector pME18S [23], designated pME18S/FLAG-CM2. A series of CM2 cytoplasmic tail deletion mutants were constructed as follows. Briefly, a plasmid expressing FLAG-tagged CM2 at residues 1–82 (FLAG-CM2 1–82) was constructed by PCR mutagenesis using pME18S/FLAG-CM2 as a template, +EcoRI-FLAG-M803 as a sense primer, and an antisense primer, −NotI-TTA-M1048 (5′-TATGCGGCCGC**TTA**ATCTTTTTCCATCGAG-3′ [NotI site, underlined; the stop codon, bold, was followed by the sequence corresponding to positions 1048–1033]). The PCR products were digested with EcoRI and NotI, and then cloned into the EcoRI and NotI sites of pME18S, designated pME18S/FLAG-CM2 1–82. pME18S expressing FLAG-tagged CM2 deletion mutants composed of residues 1–72, 1–62, 1–52, and 1–47 were constructed as described above, except that the sequences of the antisense primers were – NotI-TAA-M1018–1001 (5′-TATGCGGCCGC**TTA**AATTGTGGTCTTTATATC-3′), – NotI-TAA-M988–972 (5′-TATGCGGCCGC**TTA**CTCCCATCTGCCGAGCA-3′), – NotI-TAA-M958–944 (5′-TATGCGGCCGC**TTA**AAGTTCAATTATGAT-3′), and – NotI-TAA-M943 (5′-TATGCGGCCGC**TTA**TTTTACTAATAAATACAAC-3′), respectively. For alanine-scanning analysis, a series of plasmids expressing FLAG-tagged CM2 cytoplasmic tail mutants that contained consecutive alanine substitutions were generated by inverse-PCR using a KOD-plus Mutagenesis Kit (TOYOBO). The primer sequences are available upon request. The sequences of all constructs described above were verified by sequencing.

### 2.4. Immunoblotting of COS-1 cells expressing CM2 cytoplasmic tail mutants

COS-1 cells were transfected with plasmids expressing CM2 cytoplasmic tail mutants as described previously [5]. The cells were incubated at 37 °C, lysed at 48 h post-transfection, and resolved by SDS-PAGE on 17.5% gels containing 4 M urea under reducing or non-reducing conditions, followed by immunoblotting using monoclonal anti-FLAG M2 (SIGMA) as a primary antibody and goat anti-mouse IgG (H+L)-HRP conjugate (BIORAD) as a secondary antibody for deletion mutants, or anti-GST/CM2 serum as a primary antibody and HRP-conjugated goat anti-rabbit Ig's (BioSource International) as a secondary antibody for substitution mutants, as described previously [15]. As an internal control, α-tubulin was detected using mouse anti-α-tubulin (Santa Cruz Biotechnology) as a primary antibody and goat anti-mouse IgG (H+L)-HRP conjugate (BIORAD) as a secondary antibody. The proteins were detected by an enhanced chemiluminescence Western blotting analysis system (GE Healthcare, Piscataway, NJ) according to the manufacturer's instructions. Band intensities were measured using ImageJ 1.38 software (Wayne Rasband; National Institute of Health [http://rsb.info.nih.gov/ij/]).

### 2.5. Indirect immunofluorescence

Each plasmid expressing a CM2 cytoplasmic tail mutant was transfected into COS-1 cells. The transfected cells were fixed with 4% paraformaldehyde at 48 h post-transfection and stained with monoclonal anti-FLAG M2 (SIGMA) as a primary antibody and Alexa Fluor 488 goat anti-mouse IgG (H+L) (Molecular Probes) as a secondary antibody. COS-1 cells transfected with pME18S alone were immunostained as described above and shown as a control.

## 3. Results and discussion

### 3.1. Expression of CM2 cytoplasmic tail deletion mutants

**Fig. 1:**
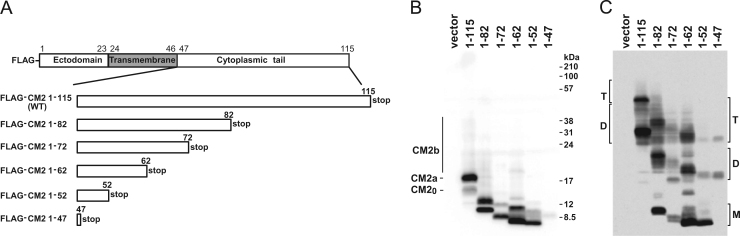
Construction of CM2 cytoplasmic tail deletion mutants and Western blot analysis. (A) FLAG-CM2 1–115 contains full-length CM2 amino acid residues 1–115. FLAG-CM2 1–82, 1–72, 1–62, 1–52 and 1–47 contain 33-, 43-, 53-, 63-, and 68-amino-acid deletions from the C-terminus, respectively. Each plasmid expressing the FLAG-tagged CM2 deletion mutants was transfected into COS-1 cells. pME18S alone was transfected as a negative control (lane vector). At 48 h post-transfection, the CM2 mutants expressed in the cells were separated by electrophoresis under reducing (B) or non-reducing (C) conditions and detected by Western blotting using monoclonal anti-FLAG M2. The tetramer (T) and dimer (D) of FLAG-CM2 1–115, as well as the T, D and monomer (M) of the CM2 cytoplasmic tail deletion mutants are indicated at the left and the right of (C), respectively.

To investigate the effects of the cytoplasmic tail of CM2 on its biochemical properties, we constructed a series of transient expression vectors encoding CM2 mutants with various deletions from the C-terminus (Fig. 1A). COS-1 cells were transfected with these vectors, lysed at 48 h post-transfection, and resolved by electrophoresis under reducing conditions, followed by Western blotting using an anti-FLAG monoclonal antibody. Representative data for six independent experiments are shown in Fig. 1B. FLAG-CM2 1–115 migrated electrophoretically as a nonglycosylated form, CM2_0_, and two glycosylated forms, CM2a and CM2b, containing a high-mannose type and a complex type carbohydrate, respectively. All CM2 deletion mutants migrated in accordance with the expected molecular size. Although the expression levels of FLAG-CM2 1–82 and 1–62 were the same as that of FLAG-CM2 1–115, the expression levels of FLAG-CM2 1–72 and 1–47 was considerably smaller than that of the wild type, with that of FLAG-CM2 1–47 being particularly low. The expression of FLAG-CM2 1–52 was slightly smaller than that of the wild type. These results suggest that amino acid residues in the regions including residues 73–82 and 48–62 may affect the level of CM2 expression. Next, the cells expressing CM2 mutants were disrupted in the presence of 50 mM iodoacetamide, as described previously [7], [10], and separated by electrophoresis under non-reducing conditions, followed by Western blotting (Fig. 1C). FLAG-CM2 1–115 was detected as CM2a and CM2b, and most of the molecules migrated as dimers and tetramers, as shown previously [7]. All CM2 cytoplasmic tail deletion mutants, including FLAG-CM2 1–47, formed tetramers, indicating that the cytoplasmic tail is not essential for the oligomerization of CM2. However, a considerable number of monomer CM2 molecules were detected among the cytoplasmic deletion mutants FLAG-CM2 1–82, 1–72, 1–62, and 1–52, suggesting that the cytoplasmic tail modulates the efficiency of CM2 oligomerization. A similar phenomenon; i.e., that mutations in the cytoplasmic domain affect oligomerization, has been demonstrated in the vesicular stomatitis virus G protein, paramyxovirus HN protein, and paramyxovirus SV5 fusion (F) protein [24], [25], [26].

### 3.2. Cell surface expression of CM2 cytoplasmic tail deletion mutants

**Fig. 2:**
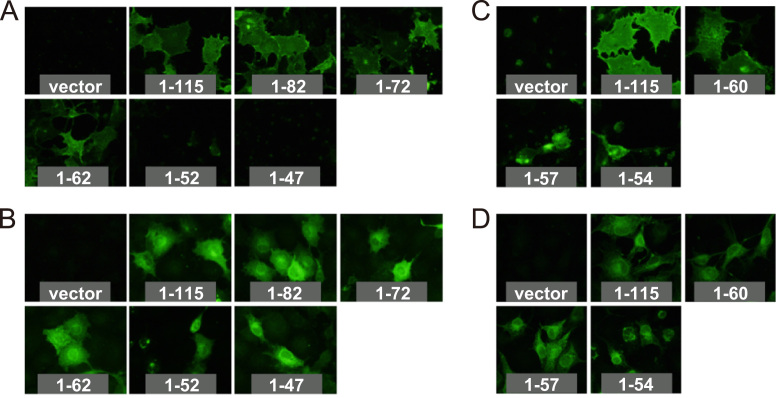
Cell surface expression of CM2 cytoplasmic tail deletion mutants. Each plasmid expressing the FLAG-tagged CM2 deletion mutants (Fig. 1A), or pME18S alone as a control (vector), was transfected into COS-1 cells. The cells were fixed with 4% paraformaldehyde at 48 h post-transfection, subsequently untreated (A) or treated with 0.5% Triton X-100 (B), and stained with monoclonal anti-FLAG M2 as a primary antibody and Alexa Fluor 488 goat anti-mouse IgG (H+L) as a secondary antibody. The cells expressing CM2 cytoplasmic deletion mutants, FLAG-CM2 1–60, 1–57, and 1–54, composed of residues 1–60, 1–57 and 1–54, respectively, were fixed, untreated (C) or treated with Triton X-100 (D), and stained as described above.

To examine the effect of the cytoplasmic tail on the cell surface expression of CM2, COS-1 cells were transfected with plasmids expressing the wild type or each of the CM2 cytoplasmic tail deletion mutants, FLAG-CM2 1–82, 1–72, 1–62, 1–52 and 1–47, fixed with 4% paraformaldehyde at 48 h post-transfection, and analyzed by indirect immunofluorescence with the anti-FLAG monoclonal antibody (Fig. 2A). The cells expressing FLAG-CM2 1–82, 1–72, and 1–62 showed bright surface staining similar to that observed for the wild type, suggesting that residues 63–115 are not essential for the transportation of CM2 to the cell surface, and that the 16 cytoplasmic amino acids at residues 47–62 are sufficient for the cell surface expression of CM2. However, no signals were detected on the surface of the cells expressing FLAG-CM2 1–52 and 1–47, suggesting that the region from residues 53–62 may contain residues necessary for CM2 transportation to the cell surface. To examine the intracellular expression of CM2 mutants in transfected cells, the cells were permeabilized with Triton X-100 after fixation and analyzed by immunofluorescence as described above (Fig. 2B). There was no significant difference in the intracellular expression between FLAG-CM2 1–115 and the deletion mutants.

To further examine which of the residues between 53 and 62 are required for cell surface expression, we constructed CM2 cytoplasmic tail deletion mutants FLAG-CM2 1–60, 1–57, and 1–54, which included a 14-, 11-, and 8-aa cytoplasmic tail, respectively, and analyzed their cell surface expression. Representative data for five independent immunofluorescence experiments are shown in Fig. 2C. FLAG-CM2 1–60 showed the same level of cell surface expression as did the wild-type FLAG-CM2 1–115. However, no cell surface expression was detected for FLAG-CM2 1–57 or 1–54, although aberrant granular signals were detected in some shrunken cells. These data suggest that the C-terminal residues 61–115 are not essential for the cell surface expression of CM2, and that a 14-aa, but not an 11-aa, cytoplasmic tail is sufficient for cell surface expression. Immunofluorescence after permeabilization with Triton X-100 showed that there was no significant difference in the intracellular expression levels between the wild-type FLAG-CM2 1–115 and the deletion mutants (Fig. 2D). It was also shown that the wild type was detected in the periphery of the cytoplasm as well as in the perinuclear region, whereas FLAG-CM2 1–47, 1–52 (Fig. 2B) and 1–54 (Fig. 2D) were all localized predominantly in the perinuclear region, a finding which also suggests impaired intracellular transport. The finding that the FLAG-CM2 1–47 and 1–52 mutants were not detected at the cell surface (Fig. 2A) may be partly explained by the low expression level of the protein as Western blotting demonstrated that FLAG-CM2 1–47 and 1–52 have lower intracellular expression levels (Fig. 1B).

### 3.3. Properties of CM2 cytoplasmic tail mutants possessing alanine substitutions

**Fig. 3:**
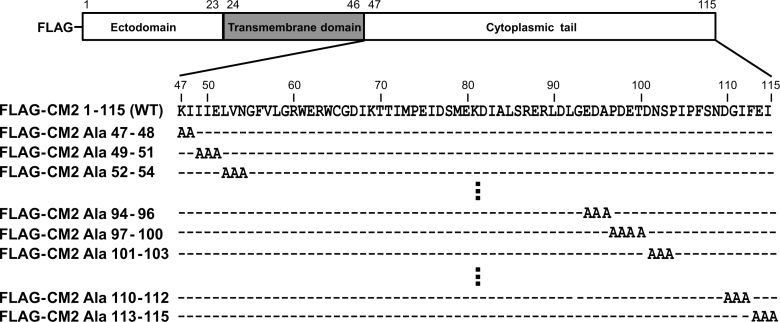
Construction of CM2 cytoplasmic tail mutants possessing alanine substitutions. The amino acid sequences of the wild-type CM2 cytoplasmic tail are shown in the upper row. All constructs contain a FLAG tag at the N-terminus. Each of the mutants FLAG-CM2 Ala 47–48 to 113–115 contains three consecutive alanine substitutions at the indicated positions, except for FLAG-CM2 Ala 47–48 and 97–100, which contain two and four consecutive alanine substitutions, respectively.

**Fig. 4:**
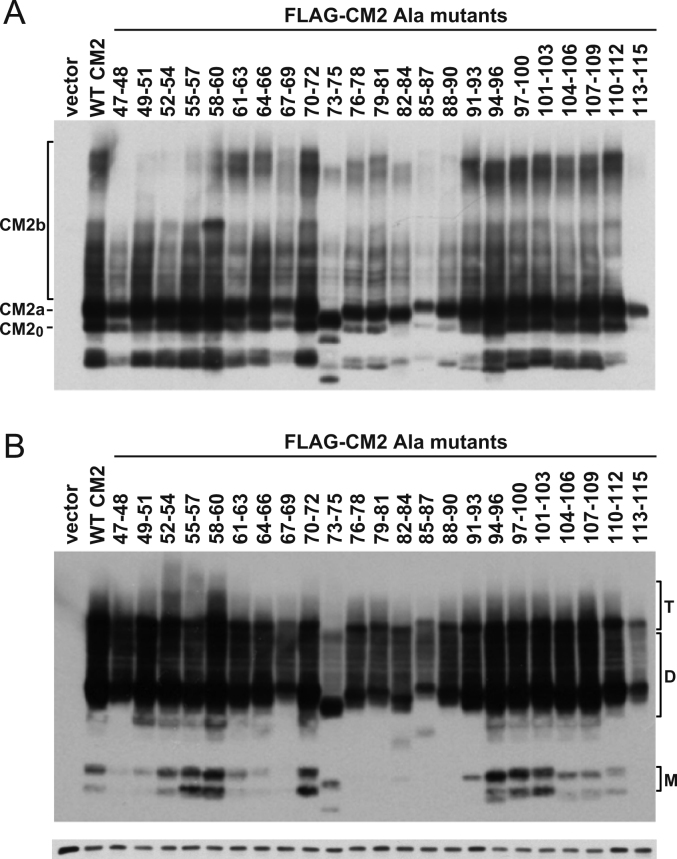
Expression of CM2 cytoplasmic tail mutants possessing alanine substitutions. Each plasmid expressing the FLAG-tagged CM2 substitution mutants (lanes 47–48 to 113–115), FLAG-CM2 1–115 (lane WT CM2), or pME18S alone as a control (lane vector) was transfected into COS-1 cells. At 48 h post-transfection, the CM2 mutants expressed in the cells were separated by electrophoresis under reducing (A) or non-reducing (B) conditions and detected by Western blotting using anti-GST/CM2 serum. The lowest panel shows α-tubulin as an internal control.

**Fig. 5:**
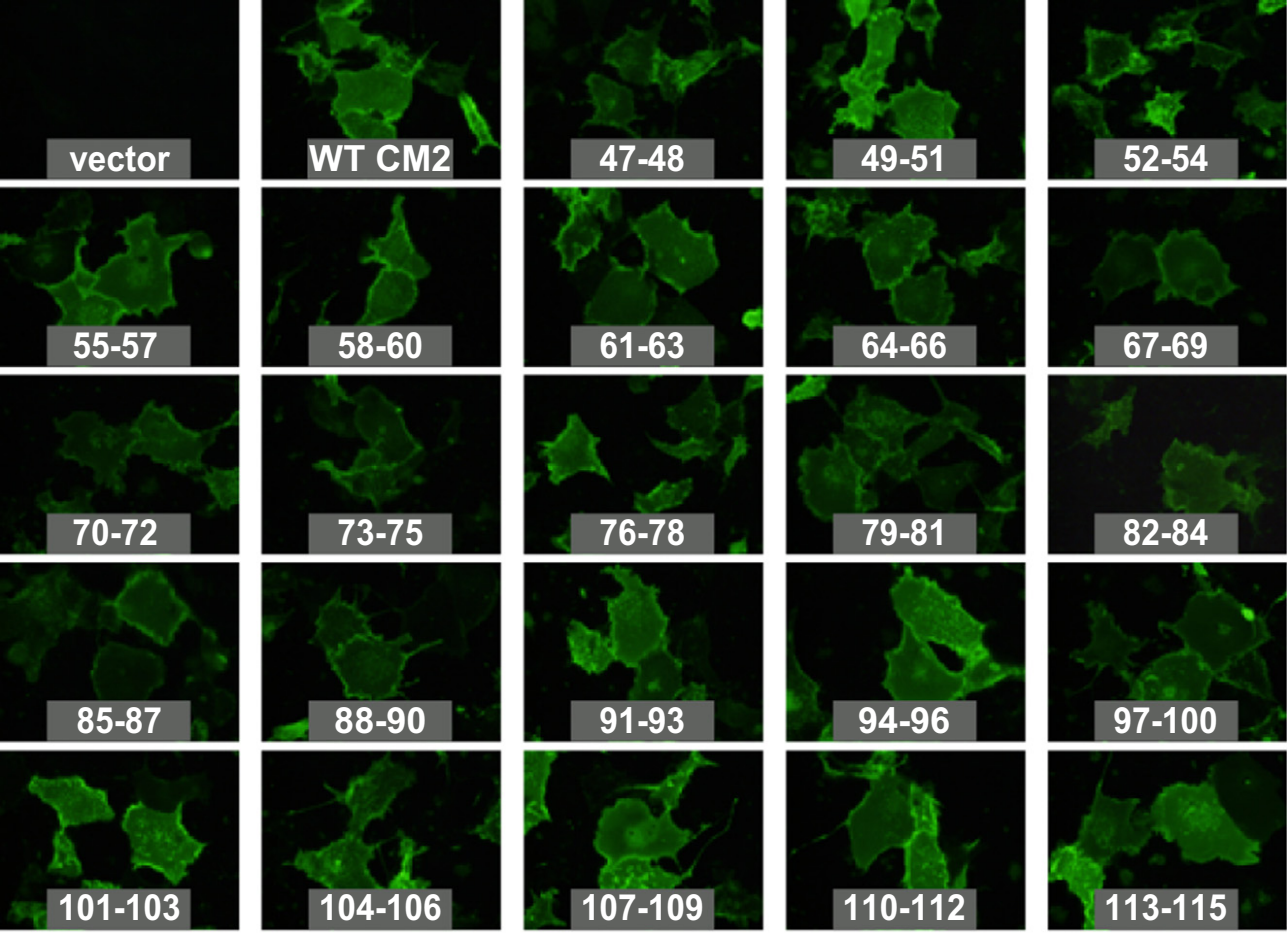
Cell surface expression of CM2 cytoplasmic tail mutants containing alanine substitutions. Each plasmid expressing the FLAG-tagged CM2 substitution mutants (47–48 to 113–115), FLAG-CM2 1–115 (WT CM2), or pME18S alone as a control (vector) was transfected into COS-1 cells. The cells were fixed with 4% paraformaldehyde at 48 h post-transfection and analyzed by immunofluorescence as described in Fig. 2A.

To further characterize the effects of the CM2 cytoplasmic tail on its biochemical properties, we generated a series of CM2 mutants by introducing consecutive alanine substitutions along the CM2 cytoplasmic tail at residues 47–115 (Fig. 3). We first analyzed the expression levels of the alanine-substituted mutants by Western blotting. The cells expressing CM2 mutants were lysed at 48 h post-transfection, resolved by electrophoresis under reducing (Fig. 4A) or non-reducing conditions (Fig. 4B), and analyzed by Western blotting using anti-GST/CM2 serum. Representative data for five independent experiments are shown in Fig. 4A. The expression levels of FLAG-CM2 Ala 47–48, 67–69, 73–75, 76–78, 79–81, 82–84, 85–87, 88–90 and 113–115 were all reproducibly much smaller than that of the wild type, suggesting that residues 47–48, 67–69, 73–90 and 113–115 are involved in the expression of CM2. The ratio of CM2b/CM2a was drastically reduced in FLAG-CM2 Ala 47–48, 52–54, 73–75 and 113–115, suggesting that residues 47–48, 52–54, 73–75 and 113–115 are involved in the conversion of CM2a into CM2b, which is accompanied by the maturation of carbohydrates from a high-mannose type to a complex type [7]. Since it is generally accepted that the maturation of N-linked carbohydrates from a high-mannose type to a complex type is accomplished in the Golgi apparatus [27], it is conceivable that residues 47–48, 52–54, 73–75 and 113–115 may be involved in the transport of CM2 from the endoplasmic reticulum (ER) to the Golgi apparatus. These data are in agreement with those from a previous report showing that mutations in the cytoplasmic domain of the influenza A virus hemagglutinin (HA), vesicular stomatitis virus G protein and paramyxovirus hemagglutinin-neuraminidase (HN) protein affect the intracellular transport of the proteins from the ER to the Golgi apparatus [24], [25], [28]. By contrast, nonglycosylated CM2 was also shown to be expressed at the cell surface [8], [16]. Therefore, we could not rule out the possibility that the CM2a or CM2_0_ of these CM2 cytoplasmic tail mutants may also be transported to the Golgi apparatus and the cell surface as all substitution mutants were expressed on the cell surface (Fig. 5). If this is the case, the inhibition of CM2a conversion to CM2b may be ascribed to an impairment in the access of N-glycosylation-processing enzymes or oligosaccharide transferases to CM2 molecules, as it is possible that mutations in the cytoplasmic tail cause conformational changes to its ectodomain, which possesses the N-glycosylation site. This idea appears to be supported by a previous report that found that mutations in the cytoplasmic domain of the Lassa virus glycoprotein caused conformational changes to its ectodomain, thereby limiting the accessibility of the ectodomain to the protease present in the ER [29]. To determine whether mutations in the cytoplasmic tail of CM2 cause conformational changes to its ectodomain, we plan to generate monoclonal antibodies against the CM2 ectodomain in the future.

Representative data for four independent Western blotting experiments after SDS-PAGE under non-reducing conditions are shown in Fig. 4B. All alanine-substituted mutants formed dimers and tetramers, suggesting that the cytoplasmic tail is not essential for CM2 oligomerization. This finding is consistent with the fact that the CM2 cytoplasmic tail deletion mutant FLAG-CM2 1–47, which lost virtually all of its cytoplasmic tail, also formed tetramers (Fig. 1C). However, the efficiency of FLAG-CM2 Ala 73–75 tetramer formation was lower than that of the wild type and other alanine-substituted mutants. These data raise the possibility that residues 73–75 may regulate CM2 oligomerization efficiency.

We next analyzed the cell surface expression of the CM2 substitution mutants by immunofluorescence (Fig. 5). All mutants were expressed on the cell surface at the same level as the wild type, suggesting that no specific cytoplasmic residues are required for CM2 transportation to the cell surface. This is consistent with our previous reports that Ala substitutions at residue 65 (acylation site) as well as residues 78, 103 and 108 (candidate sites for phosphorylation) did not affect the cell surface expression of CM2 [9], [18].

To determine whether the above data showing that consecutive alanine substitutions at residues 47–48, 67–69, 73–90 and 113–115 reduced the amount of expressed CM2 mutants could be ascribed to their instability, pulse-chase experiments were performed with COS-1 cells transfected with plasmids expressing CM2 alanine-substituted mutants, FLAG-CM2 Ala 47–48, 67–69, 73–75, 79–81, 82–84, 85–87 and 113–115, as described previously [7]. The cells were pulse-labeled with [^35^S]methionine for 30 min at 48 h post-transfection. Either immediately after a pulse or a subsequent chase of 2 h, the cells were disrupted in RIPA buffer and immunoprecipitated with anti-GST/CM2 serum. The immunoprecipitates were analyzed by SDS-PAGE on 17.5% gels containing 4 M urea under reducing conditions and processed for fluorography (data not shown). Densitometric analysis of the representative data for two independent experiments showed that the ratios of the total amount of CM2 proteins after a 2-h chase to that after a 30-min pulse in the cells expressing wild-type CM2, FLAG-CM2 Ala 47–48, 67–69, 73–75, 79–81, 82–84, 85–87, and 113–115 were 0.87, 0.46, 0.53, 0.40, 0.74, 0.74, 0.63, and 0.92, respectively. The total amount of CM2 proteins was reduced during the chase in the cells expressing wild-type CM2; however, the degree of reduction was more prominent in the cells expressing FLAG-CM2 Ala 47–48, 67–69, 73–75, 79–81, 82–84 and 85–87, suggesting that residues 47–48, 67–69, 73–75 and 79–87 may play a role in CM2 stability and, thus, are involved in its expression level. By contrast, FLAG-CM2 Ala 113–115 showed a level of stability similar to that of the wild-type CM2. Therefore, the mechanism underlying the low level of FLAG-CM2 Ala 113–115 expression remains unclear. Furthermore, the data demonstrated that the tetramerization of FLAG-CM2 Ala 73–75 was impaired (Fig. 4B), indicating that FLAG-CM2 Ala 73–75 may take an aberrant conformation, thereby causing it to become unstable. Therefore, it is possible that 73-MPE-75 plays an important role in ensuring proper conformation, providing CM2 with both stability and suitable access to enzymes involved in N-glycosylation during its intracellular transport. However, we could not rule out the possibility that the reason why the tetramerization of FLAG-CM2 Ala 73–75 was impaired (Fig. 4B) was partly that we changed a proline residue, which tends to introduce specific conformational constraints, and a charged glutamic acid residue, which is often involved in salt-bridges. By contrast, Western blot analysis of influenza A virus M2 protein demonstrated that triple-scanning alanine mutations in the cytoplasmic tail at residues 46–69 did not alter the oligomerization potential of M2 when alanine substitutions were introduced at Glu56, Pro63, Glu66, and Pro69 [30].

The results of this study suggest that certain amino acid sequences or the length of the CM2 cytoplasmic tail are involved in the proper conformational maturation, stability, expression level and intracellular transport of CM2. However, it remains to be determined whether these CM2 cytoplasmic tail mutations affect influenza C virus replication.
